# Hardware Implementation of Multiple Fan Beam Projection Technique in Optical Fibre Process Tomography

**DOI:** 10.3390/s8053406

**Published:** 2008-05-23

**Authors:** Ruzairi Abdul Rahim, Mohd Hafiz Fazalul Rahiman, Lai Chen Leong, Kok San Chan, Jon Fea Pang

**Affiliations:** 1 Control & Instrumentation Engineering Department, Faculty of Electrical Engineering, Universiti Teknologi Malaysia, 81310 Skudai, Johor Bahru, Malaysia; 2 School of Mechatronic Engineering, Universiti Malaysia Perlis, 02600 Arau, Perlis, Malaysia; E-Mail: hafiz@unimap.edu.my

**Keywords:** optical tomography, Fan Beam, projection, optical fibres

## Abstract

The main objective of this project is to implement the multiple fan beam projection technique using optical fibre sensors with the aim to achieve a high data acquisition rate. Multiple fan beam projection technique here is defined as allowing more than one emitter to transmit light at the same time using the switch-mode fan beam method. For the thirty-two pairs of sensors used, the 2-projection technique and 4-projection technique are being investigated. Sixteen sets of projections will complete one frame of light emission for the 2-projection technique while eight sets of projection will complete one frame of light emission for the 4-projection technique. In order to facilitate data acquisition process, PIC microcontroller and the sample and hold circuit are being used. This paper summarizes the hardware configuration and design for this project.

## 1. Process Tomography Overview

The widespread need for direct analysis of the internal characteristics of process plants in order to improve the design and operation of equipment has made process tomography a main research activity within the industrial instrumentation. Originated from the Greek words *‘tomos’* which means slice and *‘graph’* meaning picture, tomography can be defined as a picture of a slice [1]. In simple terms, tomography is an imaging technique that enables one to determine the contents of a closed system without physically looking inside it.

There are different requirements in an industrial environment than there are within a medical one: different regulations regarding for example use of ionising modalities and different speed requirements [2] Technically, Process Tomography can be described as imaging process parameters in space and time. Important flow information such as concentration measurement, velocity, flow rate, flow compositions and others can be obtained without the need to invade the process or object. As a result, cross sectional images of processes generate better online inspection, monitoring and process control -promoting improved yields and more effective utilization of available process capacity. Potentially, tomographic systems may also be an alternative approach in developing and verifying process theories and models, as well as for improving process instrumentation.

The earlier researches done by Ruzairi [3], Sallehuddin [4], Khoo [5] and Hisyamuddin [6] have shown that the optical fibre sensor is applicable in flow visualization (image reconstruction). The acquired concentration profile from the image reconstruction is needed together with the velocity profile to complete the mass flow rate estimation in a pneumatic conveying system. Basically, the principle of measurements in tomography is to obtain all possible combinations of measurements from the sensor system. The higher the measurements obtained from the sensors, the resolution of the system would be better.

By using the parallel projection, previous researches have each faced the problem of obtaining a high resolution of their system. This is because the parallel projection method limits the number of measurements to the number of sensors being used. In his research, Chan [7] has implemented the switch-mode fan beam projection technique to obtain flow visualization using LED as light source but resolution and the number of sensors in his system is limited by the physical size of the LED emitters. Thus, this research will focus in implementing the multiple fan beam method using optical fibre sensors to increase both the number of sensors and number of measurements in order to obtain a system with high resolution.

## 2. Introduction to the Hardware System

**Figure 1.**
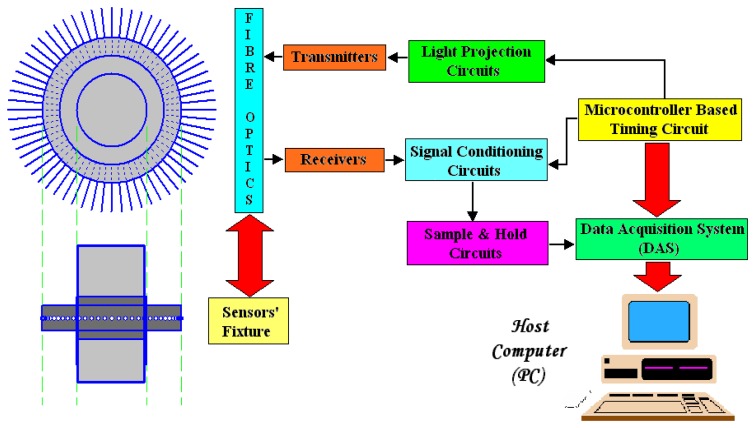
Topology of the hardware construction.

A typical Optical Fibre Process Tomography (OFPT) system consists of the sensor's array, signal control and conditioning circuit, data acquisition system (DAS) and also the display unit, namely the computer. Topology of the constructed hardware system in this research is illustrated in Figure 1.

By referring to Figure 1, the micro controller is used to control the duration of light projection, sample and hold digital input and data acquisition system (DAS) synchronization signals. Through the fibre optics, photodiodes detect the physical signals (light beams) from the transmitters. In the signal conditioning circuit, the physical signals are being converted into voltage readings and then amplified. The analogue signals go through the sample and hold circuit before being transferred into DAS. DAS then converts the analogue signals into digital signals. These digital signals are being sent to the PC for image reconstruction.

By referring to Figure 1, the micro controller is used to control the duration of light projection, sample and hold digital input and data acquisition system (DAS) synchronization signals. Through the fibre optics, photodiodes detect the physical signals (light beams) from the transmitters. In the signal conditioning circuit, the physical signals are being converted into voltage readings and then amplified. The analogue signals go through the sample and hold circuit before being transferred into DAS. DAS then converts the analogue signals into digital signals. These digital signals are being sent to the PC for image reconstruction.

## 3. Selection of Emitters and Receivers

Emitters and receivers are the main optical sensors that must be carefully chosen to satisfy the characteristics and requirements of the hardware system. According to Chan [7], for a system which implements the switch-mode fan beam projection, the emitter must have a very fast setting time when driven by a pulse current while the receiver must have fast transient characteristic when exposed to the switched light sources. The selection of the sensors is based on the specified requirements such as size of the emitting area, angular spread of the emitted light, reliability, physical size, dynamic response and costs of the light source.

For the emitters, three types of optical devices are being considered and they are the light emitting diode (LED), infrared (IR) and laser diodes. Although the laser diodes have a fast operational speed, the LED is generally user friendly and is certainly more cost effective when compared with laser diodes. Besides that, the output power of the LED is linearly proportional to driving the current while laser diodes have an output power which is proportional to current above the threshold.

Linearity is an important characteristic to light sources in analog applications which is emphasized in the implementation of the OFPT sensors. Based on the comparisons of the LED and laser diodes in terms of linearity, costs and it is found that the LED is a better choice of emitter for this project.

However, there is a weakness in LED to be used as transmitter because the light of LED is visible light with the wavelength in between 380-700 nm and therefore results in the tomography sensor designed is easily getting noise from the surrounding environment light source [8]. Most lights sources that we use daily are white or visible lights such as the incandescent lamp (light bulb) and fluorescent light which have a peak of radiant power at 550 nm that can simply affect the light received by the photo-receivers.

The most suitable part of the spectrum of light which is suitable to be selected as light source for this project is the infrared. Generally, the wavelength of the infrared LED lies in between 700nm to 1100nm, thus potentially can safeguard the tomography sensor from being affected by visible light.

The selected transmitter used is the SFH484-2 GaAIA infrared emitter with its peak wavelength at 880n. The small radiation angle is necessary because the emitting area needed for the infrared to be coupled with the fibre optics is small and narrow.

Meanwhile, the main requirements to choose the photo-receivers is to select a photo-receiver with high sensitivity, fast switching time (taking into account the transient/rise and fall time), cost effective and most compatible with the selected infrared emitter. Phototransistors generally have a slower response than photodiodes [9] and linearity of the phototransistor is over a much narrower range than a photodiode. Rise time of the phototransistor is poor due to the combined capacitance of the B/E and C/E junctions and the lifetimes of the carriers in the depletion region of the junction. Based on the need of a fast response and high sensitivity, the photodiode is selected as the photo-sensor for this hardware system instead of the phototransistor.

Basically, all the photodiode models have a fast switching time of 5ns and they have the same diameter. Thus, selection is mainly based on the price and also the spectral range. The first model, SFH203P is apparently the cheapest but a main concern is that if has a very wide spectral range from 400nm to 110nm. As stated earlier, the visible light has a range of 380nm to 700nm, thus this photodiode performance might be influenced by the visible light from the environment. The second model, SFH203PFA has a narrow spectral range but the price is too expensive. The most reasonably priced and has the most agreeable spectral range is the SFH213-FA photodiode and this will be selected to match with the infrared transmitter. Besides that, the SFH213-FA has a fast transient time which can reduce the signal setting time [10].

### 3.1 Preparation of Optical Fibres

**Figure 2.**
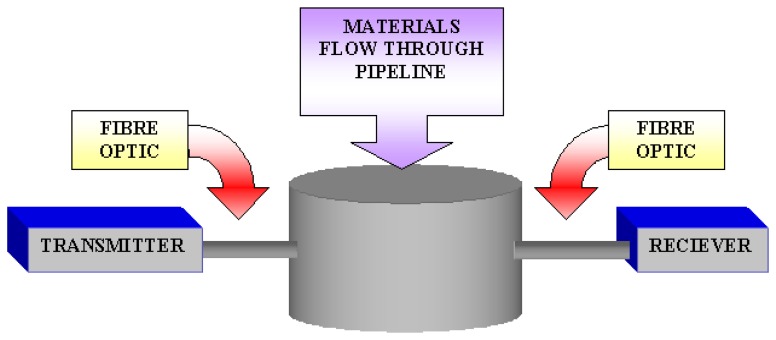
Fibre optics configurations.

In the area of tomographic imaging, an initial investigation into using fibres optic as measurement sensors in pneumatic conveying was started in Sheffield Hallam University [3]. In using optical fibre for tomography imaging, the basic optical transmitter converts electrical input signals into modulated light for transmission over an optical fibre. Also, the light beam from the transmitter is being received by the receiver via the fibre optic. This configuration is being illustrated in Figure 2.

**Figure 3.**
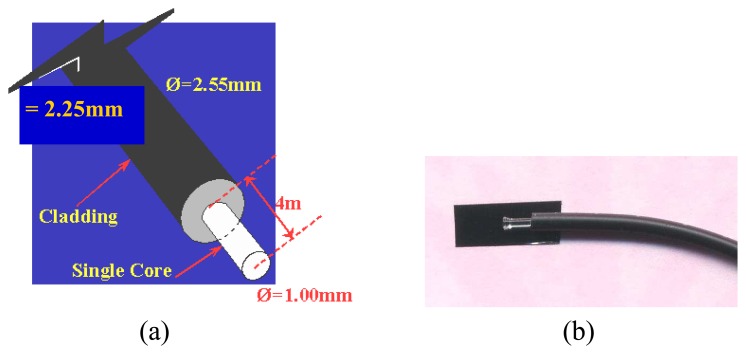
**(a)** Optical fibre end termination **(b)** Drawing Actual photograph

With regard to its small physical size, it is believed that using fibre optic will allow a higher number of optical sensors to be installed, thus achieving high-resolution measurement in optical tomography. It is also said that the optical fibre sensors provide wide bandwidth which enables measurements to be performed on high speed flowing particles [4]. As stated earlier in this paper, the optical fibres are used together with the selected transmitters and receivers. The choice of using single core polymer cable fibre optic (with core diameter at 1.00mm and overall diameter at 2.25mm) instead of the fibres made of glass is because the former is more affordable, easier to install [11] and since the core is made of plastic instead of glass, terminating the cable will be easier [3]. Figure 3 shows the fibre optic after treatment.

**Figure 4.**
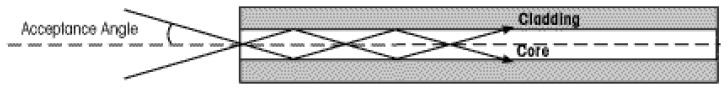
The acceptance angle for optical fibre.

The fibre optic has a numerical aperture of 0.47 and acceptance angle of 28 degrees as stated in the data sheet. The numerical aperture determines the acceptance cone of the fibre [12]. Equation1 gives us the formula to calculate the numerical aperture and Figure 4 shows the acceptance angle of an optical fibre. The total receiving angle for the fibre optic is two times the acceptance angle and in this case, it is 56 degrees.

(1) $$NA=\sin\theta_{A}$$

Whereby:

*NA* = numerical aperture of the fibre optic.
*θ_A_* = acceptance angle of the fibre optic.

**Figure 5.**
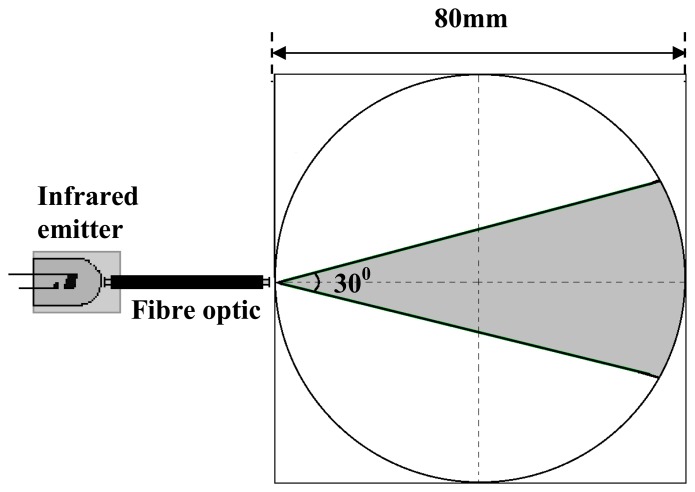
Determining emission angle.

Unlike in the application of optical fibre sensors in parallel beam projection, the emission beam should not concentrate in a straight line. Instead, the emitted fan beam should have a transmission angle. Preliminary testing shows that the maximum achievable emission angle for the fibre optic transmitter is about 30°, after the fibre optic is being lensed. There are 32 fibre optic transmitters that are being used in this research; thus in order to make sure that the emission angle is approximately the same, each of the fibre optic emission angles is being tested experimentally as illustrated in Figure 5.

### 3.2 Fibre optic coupling

**Figure 6.**
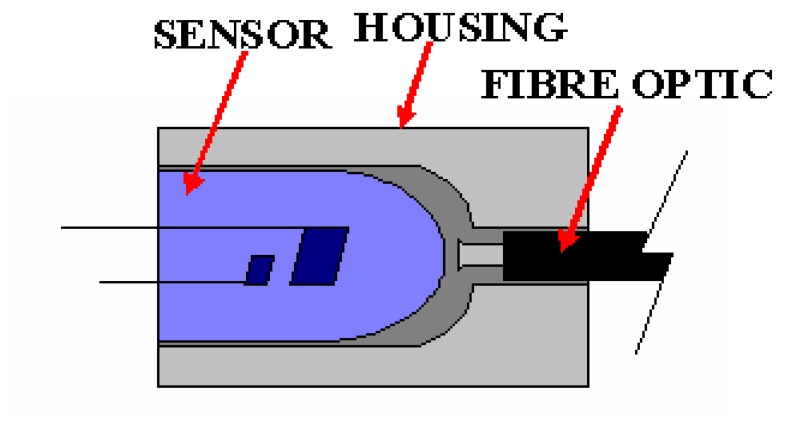
Coupling between sensor and fibre optic.

**Figure 7.**
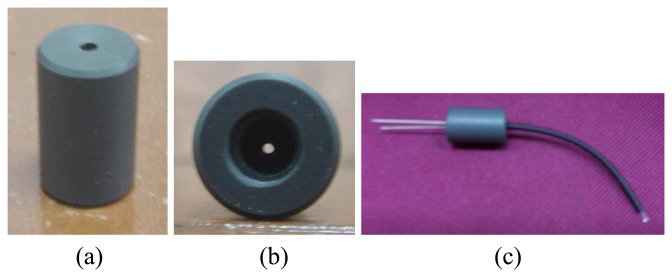
PVC housing **(a)** 3-dimensional view **(b)** Bottom view **(c)** Coupling with infrared emitter and fibre opti.

Signal loss due to improper coupling between sensors and fibre optics can cause inaccurate data acquisition. In order to avoid transmission loss when the fibre optics is coupled with the infrared emitters and photodiodes, custom-made housing is being used. The housing is made of PVC and designed as such to hold both the infrared emitters and photodiodes with fibre optics to make sure that the connection area is small and the lights can be directed straight, either from the emitters to the fibre optics or light from the fibre optics to the receivers. Figure 6 shows the coupling between the sensor and fibre optic while figure 7 shows the actual photographs of the fibre optic housing and its coupling with the sensor and fibre optic.

### 3.3 Optical Fibre Sensor Fixture Design

**Figure 8.**
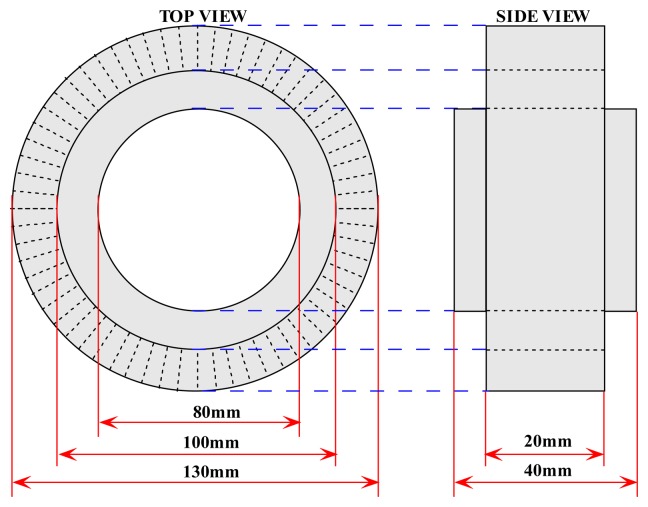
Top view and side view of the sensor fixture.

**Figure 9.**
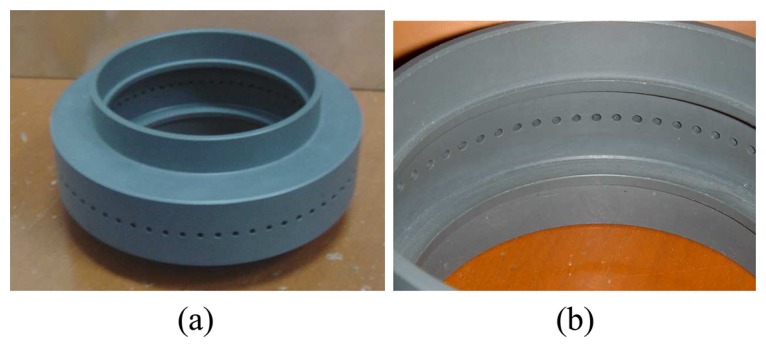
Optical fibre sensor fixture: **(a)** 3-dimensional view **(b)** Internal view

A custom-made sensor fixture is being designed to hold and support the optical fibres. The sensor fixture is also made of PVC and 64 holes (each with a diameter of 2.3mm) are being drilled along the periphery of the fixture. For a pipe with 80mm inner diameter, the diameter of the fixture peripheral that supports the fibre optics is 100mm as shown in Figure 8. Figure 9 further shows the actual photography of the optical fibre sensor fixture.

## 4. The Signal Processing Circuits

### 4.1 Infrared Projection Circuit

**Figure 10.**
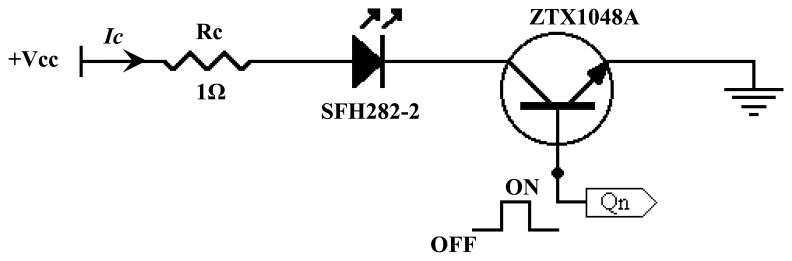
Infrared projection circuit.

Infrared projection circuit to supply current for infrared transmitters to transmit light. The infrared projection circuit used in this research is shown in Figure 10 below using the basic components of 1Ω resistor and ZTX1048A transistor.

From the above circuit, the collector current, *I_c_* can be calculated using Equation 2

(2) $$I_{c}=\frac{V_{cc}-V_{f}-V_{CE\;(\text{sat})}}{R_{c}}$$

Whereby:

*I_c_* = collector current or forward current for the projection circuit.
*V_cc_* = voltage supply which is 4.5V in this circuit.
*V_f_* = forward voltage of the SFH484-2 which is 3V as stated in datasheet.
*V_CE_*_(_*_sat_*_)_ = collector-emitter saturation voltage for the ZTX1048A transistor, which is 245mV as stated in datasheet. *R_c_* = resistor with the value of 1Ω.

Thus, with the given values, *I_c_* can be calculated as follows:

$$I_{c}=\frac{5-3-0.245}{1}=1.755A$$

From Figure 10, the point Q_n_ at the base of the transistor is connected to the decoder in the signal control circuit. The n represents the n-th decoder output pin as there are 32 individual infrared projection circuits. Base current determines the ‘on’ and ‘off’ state of the projection circuit. Positive pulse that is supplied will activate the projection circuit while negative pulse will deactivate the circuit. This operation mode is known as the pulsed mode. The emitter is operated in pulsed mode because it can handle a larger current and hence generate a greater intensity of radiation [7]. For example, for a certain infrared, if applied with continuous current, the maximum achievable forward current is 100mA while pulsed current might be able to withstand forward current up to 1A for 5ms [13].

### 4.2 Signal Conditioning Circuit

**Figure 11.**
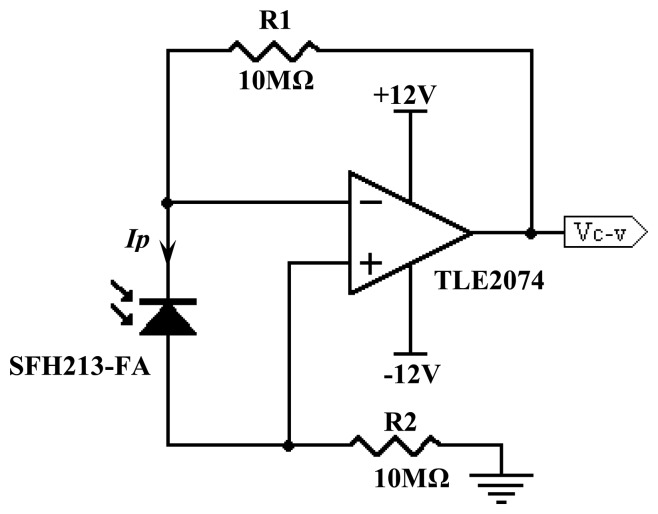
Current-to-voltage converter circuit.

Signal conditioning circuit which functions as the current to voltage converter for the physical signals received by photodiodes. The voltage readings are further amplified to an acceptable level to be observed. The principle of an optical tomography system is to investigate the light attenuation level for each detector. The signal conditioning circuit for this hardware system is divided into two stages which are the current-to-voltage converter stage and the voltage amplification stage. Figure 11 illustrates the current-to-voltage converter circuit that is used.

The current output response of the photodiode is linearly related to the incident light energy. A monitor of this current must have zero input impedance to response with no voltage across the photodiode. Zero impedance is the role of an op-amp virtual ground as high-amplifier loop gain removes voltage swing from the input [14]. In another words, main job of the op-amp is to adjust the output such that the inverting input equals the non-inverting input. This is the key to the basic current-to-voltage connection circuit as shown in Figure 11.

The output voltage of the pre-amp, Vc-v is obtained by using Equation 3.

(3) $$V_{c-v}=I_{p}\;(R_{1})$$

Whereby:

*V_c–v_* = pre-amp output voltage.
*I_p_* = photodiode current.
*R*_1_=feedback resistor of 10Ω.

According to Wong [15], the feedback resistor R_1_ determines the transimpedance, and hence the sensitivity of the amplifier. Large R_1_ increases sensitivity but at the same time reduces the amplifier's bandwidth since it contributes to the pre-amp's input load impedance.

**Figure 12.**
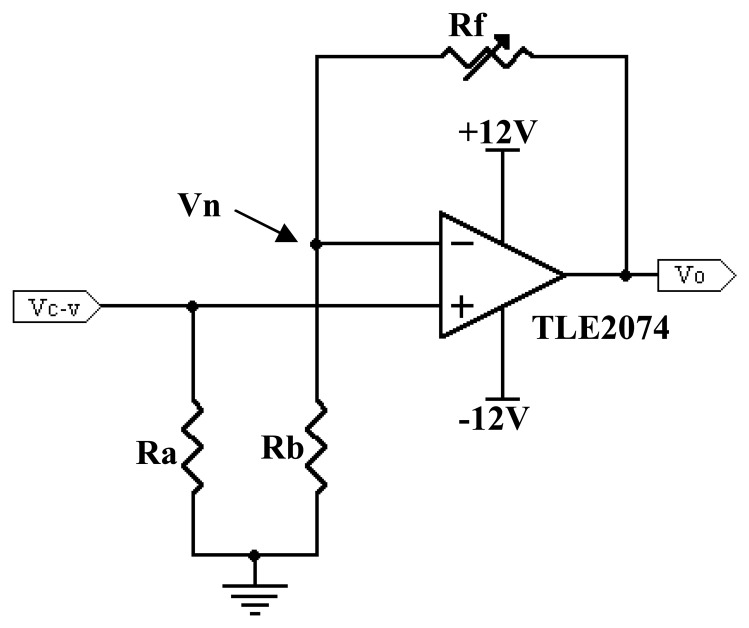
Non-inverting voltage amplifier circuit.

From the first stage of current-to-voltage converter, the pre-amp voltage will be sent to another op-amp to be amplified. Figure 12 shows the non-inverting voltage amplifier circuit [16].

The same TLE2074 op-amp is being used here in this amplifying circuit. The voltage at the inverting input *V_n_* is defined by Equation 4.

(4) $$V_{n}=\frac{V_{o}\;(R_{b})}{R_{f}+R_{b}}$$

Whereby:

*V_n_* = voltage at the inverting input.
*V_o_* = output voltage after the amplifying stage.
*R_f_* = variable feedback resistor of 500kΩ.
*R_b_* = resistor of 100kΩ

Since the differential voltage is zero, *V_n_* = *V_c_*_−_*_v_* and thus, the output voltage can be obtained as referred to Equation 5 (Boylestad and Nashelsky, 1999), with *V_o_*, *V_c_*_−_*_v_*, *R_f_* and *R_b_* parameters are as defined earlier.

(5) $$V_{o}=V_{c-v}\;\left(1+\frac{R_{f}}{R_{b}}\right)$$

In this case, the parallel combination values of *R_f_* and *R_b_* results in an approximation of 83.3kΩ. Since *R_f_* has a variable resistive value, *R_a_* is selected to be 100kΩ. This additional resistor *R_a_* is desirable because the voltage drops due to bias current to the inputs are equal and cancel out even over temperature [16]. Thus, the overall performance of the circuit is much improved.

### 4.3 Microcontroller signal controlling circuit

Previous researchers done by Ruzairi [3], Sallehuddin[4], Chan [7] and Pang [17] used the digital signal control circuit whereby each alteration to the signals needs reconstruction of the logic circuits or devices. This is found to be very troublesome and not flexible when alterations are done to the signal controls.

**Figure 13.**
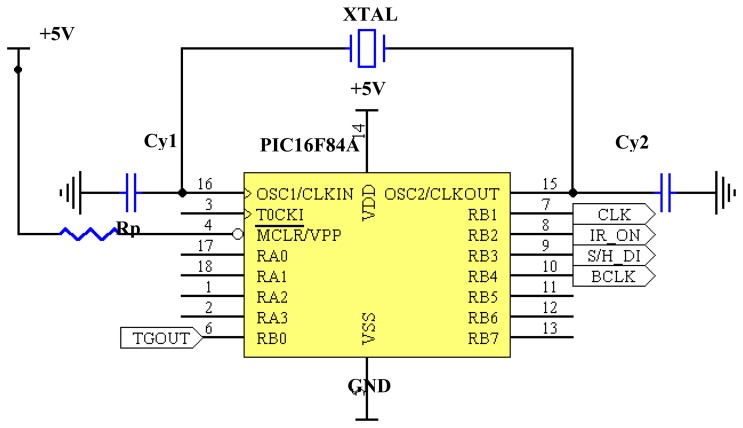
PIC16F84 circuit connection.

The main motivation to use the PIC16F84A microcontroller is because the device has sufficient requirements to support the needs of this project. In this signal controlling circuit that has been designed, the micro controller is used to control the duration of light projection, sample and hold digital input and data acquisition system (DAS) synchronization signals. The circuit connection of the microcontroller is shown in Figure 13.

With reference to the circuit connection, $\bar{\text{MCLR}}$ is the master clear or reset input and has an active low reset to the device. Usually, it is being connected via a resistor to the positive supply pole to prevent from bringing a logical zero to the $\bar{\text{MCLR}}$ pin accidentally. This resistor, whose value is selected as 4.7kΩ and its function is to keep a certain line on a logical one as a preventive, is called a pull up. Meanwhile, the XTAL is the crystal oscillator and the value of this crystal used is 2MHz with two ceramic capacitors (a value of 22pF each). For the I/O pins, RB0 functions as an input pin (TGOUT input from the DAS) while RB1, RB2, RB3 and RB4 are output pins for clock (CLK), signal to control the duration of ‘on’ and ‘off’ state of the infrareds via decoder (IR_ON), digital input control of sample and hold circuit (S/H_DI) and also the burst clock to signal DAS to start its data conversion process (BCLK).

**Figure 14.**
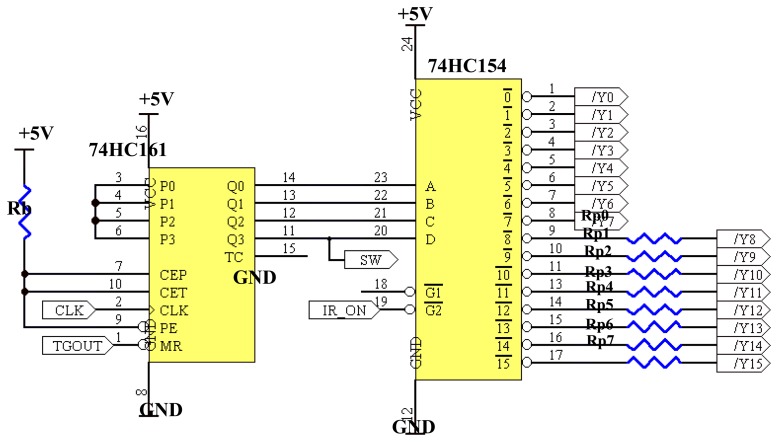
Binary counter and decoder circuit.

CLK is the heartbeat to the signal control circuit and is connected to the 74HC161 binary counter while IR_ON will determine the duration of 74HC154 decoder to activate its output to control the light projection circuit. Since the outputs of the 74HC154 have active low outputs [16], the 74HC04 Inverters is being used to toggle the decoder outputs from low to high before connecting to the base of transistors Q_n_ in the infrared projection circuits. The 74HC04 has six independent inverters [18] and since there are sixteen outputs for the decoder, a total of three inverter chips are needed. The basic connection circuit for the binary counter and the decoder is exemplified in Figure 14.

A decoder is a logic circuit that accepts a set of input that represents a binary number and activates only the output that corresponds to that input number [19]. The binary input of the decoder is controlled by the high speed, 4-bit 74HC161 binary counter and the counter is activated by the TGOUT input to $\bar{MR}$ which is the master reset for the 74HC161 [20]. The counter will stay idle unless there is a positive edge-trigger which activates it. Once activated, the CLK signal from microcontroller will drive the counter at the programmed frequency.

**Figure 15.**
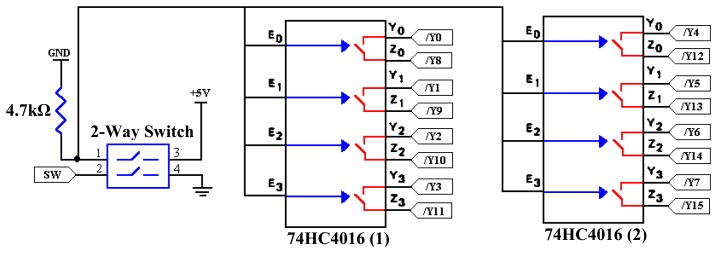
Bilateral and 2-way switch connections.

Not only the outputs of the decoder are connected to the 74HC04 inverters, they are also connected to the 74HC4016 bilateral switch. This bilateral switch is to change the hardware configuration to perform either in the 2-projection or 4-projection mode. Figure 15 shows the connections for the bilateral switches which are connected also to the 2-way switches.

The 74HC4016 has four independent analog switches [21] and has an input control to active the switch. Here, these input controls for all the bilateral switches are connected to the 2-way switch. In the 2-projection mode, both the 2-way switches are left ‘open.’ In this switch configuration, the outputs of the decoder perform as individual pins. For example, activating /Y0 will set Tx0 and Tx16 to ‘on’ and /Y8 will set Tx8 and Tx24 to ‘on’. If the 2-way switch is in ‘closed’ mode, current will flow from pin 3 to pin 1 before heading for ground. This way, all the input controls for the bilateral switches will be activated. When this happens, /Y0 will be in the same configuration as /Y8, and therefore, Tx0, Tx16, Tx8 and Tx24 will ‘on’ to satisfy the 4-projection mode. For the SW input from 74HC161 binary counter, it is referred to ground when used in 4-projection mode. This input represents the MSB which should be connected to ground because the 4-projection mode need only 3bit binary counter.

### 4.4 Sample and Hold (S/H) Circuit

The sample and hold or S/H function is one which is basic to the data acquisition and A/D conversion process. In most applications, the sample and hold is used as the “front-end” to an A/D converter in data acquisition systems [22]. In these applications, the S/H amplifier is used to store analog data which is then digitized by a relatively slow A/D converter. In this fashion, high speed or multiplexed analog data can be digitized without resorting to complex and expensive ultra-high speed A/D converters [23-24].

Basically, a sample and hold amplifier circuit has two basic and distinct operational states. In the ‘SAMPLE’ stage, an input signal is sampled and simultaneously transmitted to the output. For the ‘HOLD’ stage, the last value sampled is held until the input is sampled again. When the S/H goes into the ‘HOLD’ stage, the S/H switch opens and the voltage stored by the hold capacitor settles through the output buffer. The positive or negative bias current of the output buffer starts charging or discharging the hold capacitor. This degradation of the hold capacitor's voltage over time is known as the “droop rate” [25].

**Figure 16.**
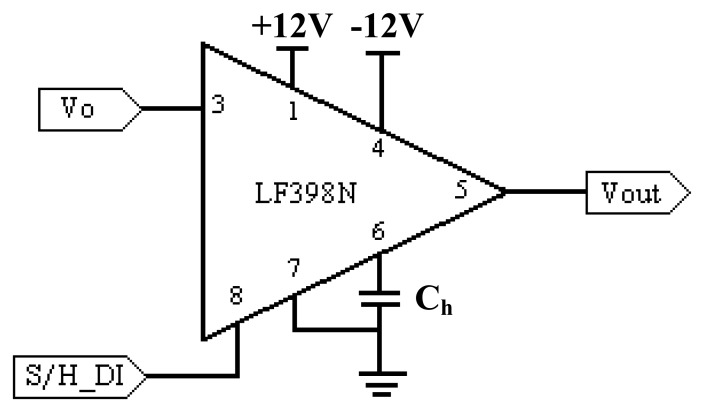
Sample and hold circuit.

The choice of hold capacitor is important as droop rate is part the major trade-offs in the selection of a hold capacitor value. The leakage of electrolytic and the transient behavior of ceramics rule them out completely in this application. The best choice is probably polypropylene, and after that polystyrene or Mylar [23]. Everything necessary for the S/H except the hold capacitor can be put on chip, so monolithic sample-and-hold circuits, like the LF398, are available and very easy to use. The S/H command is given through a digital logic level, so these circuits interface directly with logic. Besides that, the LF398 has a hold step of less than 1mV, has an acquisition time of 4μs, features high input resistance and also has a low output resistance. Based on these advantages, the LF398 is selected for this research. The S/H circuit is illustrated in Figure 16.

From Figure 16, the Vo is the analog output voltage after amplification from the signal conditioning circuit, while the SSH_DI is the digital logic signal generated by the microcontroller. C_h_ is the hold capacitor which has a value of 1.5nF and Vout is the output voltage for the sample and hold chip.

## 5. Data Acquisition Process

**Figure 17.**
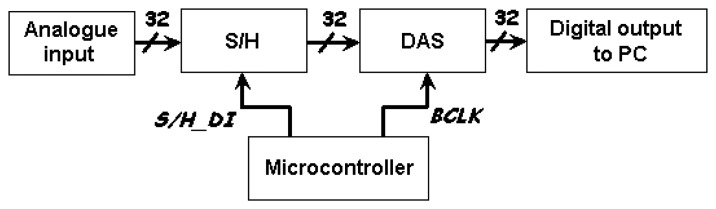
Data acquisition process.

For the purpose of converting the analogue signals from the signal conditioning circuit before the data is being processed by the computer for image reconstruction, the Keithley DAS-1802HC high speed data acquisition board has been selected. Figure 17 shows the data acquisition process system.

**Figure 18.**
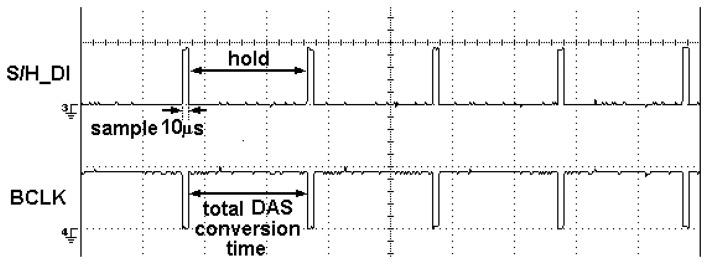
S/H_DI and BCLK signals

Analogue input from the hardware system goes through the sample and hold circuits before being sent to DAS for analogue to digital conversion. The S/H_DI sends a signal from the microcontroller to the S/H circuit to sample all output signals for a short period of 10μs and then continue to hold the sampled output signals until it receives the next rising edge. At the same time when the S/H signals are on hold, the BCLK signal will send a positive edge signal to the DAS to start data conversion as shown in Figure 18. The total duration of the data conversion time depends on the maximum burst mode clock frequency of 333 kHz in for this DAS.

**Figure 19.**
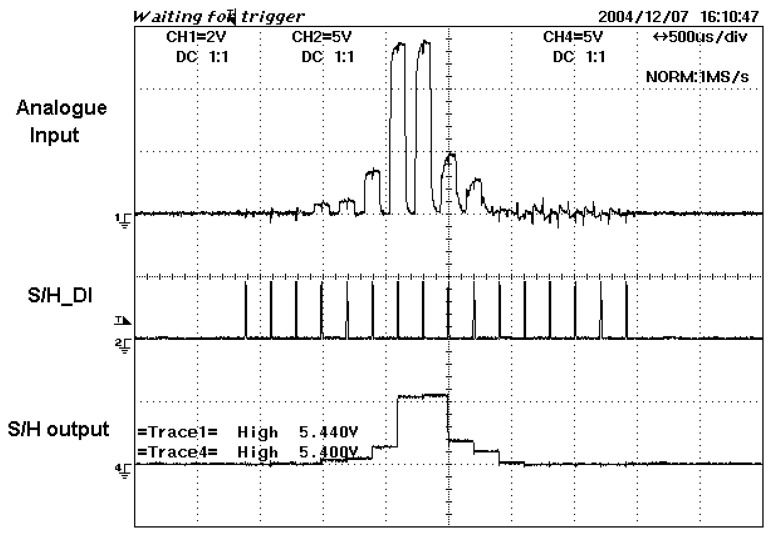
Sample and hold execution.

When there are many analogue inputs that are needed to be converted into digital outputs, the sample and hold circuits come in handy. For example, in this paper, there are 32 analogue inputs fed in parallel into the 32 channels DAS buffers. A single digital input control signal from the microcontroller will request all 32 individual sets S/H circuits to sample all the analogue signals synchronously. All the signals on-hold will be sent also in parallel to the DAS for data conversion. This will save execution time whereby all 32 analogue signals need not wait to be sampled in serial, which is sampling the 1^st^, followed by 2^nd^ signal, 3^rd^ signal until 32^nd^ analogue signals. Figure 19 illustrates an example of the analogue and digitalized S/H output signals for Channel 23.

**Figure 20.**
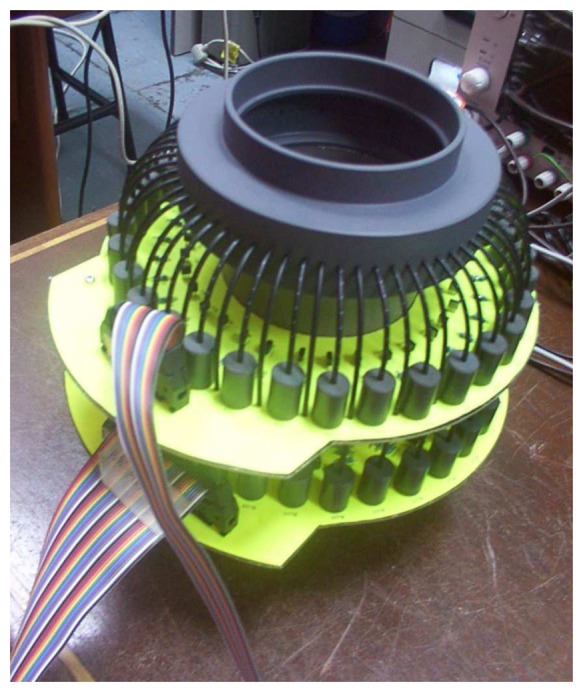
Actual photographs of the hardware system

The actual photographs for the hardware system and PCB boards are shown in Figure 20.

## 6. Results & Discussions

### 6.1 Measured Signals from Oscilloscope

The Yokogawa DL1540 4-Channel Digital Oscilloscope and Tektronik TDS3014 4-Channel Digital Oscilloscope are being used to visualize and also measure the desired waveforms or signals obtained from the hardware. Preliminary results of the hardware development, such as the response of the photo-sensors, microcontroller controlling signals, pre-amp voltages and output voltages will be presented.

#### 6.1.1 Photo-sensors

**Figure 21.**
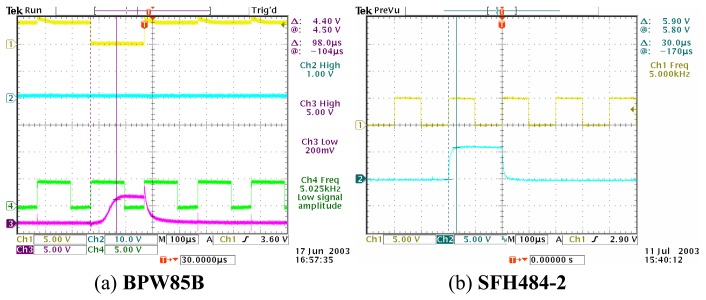
Photo-receivers' transient response.

The various selections of photo-sensors have been discussed previously. Among the topics of discussion is the comparison of the phototransistor and photodiode's performance test in order to select the most suitable photo-sensor. It has been agreed that the SFH213-FA photodiode has been chosen since it is cost effective, has a fast transient time and its spectral range is compatible with the SFH484-2 infrared emitter. However, before the research opted for photodiode as receiver, a few tests are done to proof that the phototransistor has a slower transient time when compared to the phototransistors. For comparison purposes, the BPW85B phototransistor and SFH203-FA photodiode are exposed to a pulsed light (of 5 kHz) from the SFH484-2 infrared emitter. The responses of the photo-receivers are being illustrated in Figure 21.

Obviously, the SFH484-2 photodiode (with a transient time of approximately 30μs and fall time also about 30μs) has a faster switching time than the BPW85B phototransistor (with a transient time of approximately 98μs and fall time of about 60μs). It is thus proved that the photodiode is more suitable to be used in this research compared to the phototransistors due to its fast transient and fall time.

#### 6.1.2 Microcontroller Controlling Signals

**Figure 22.**
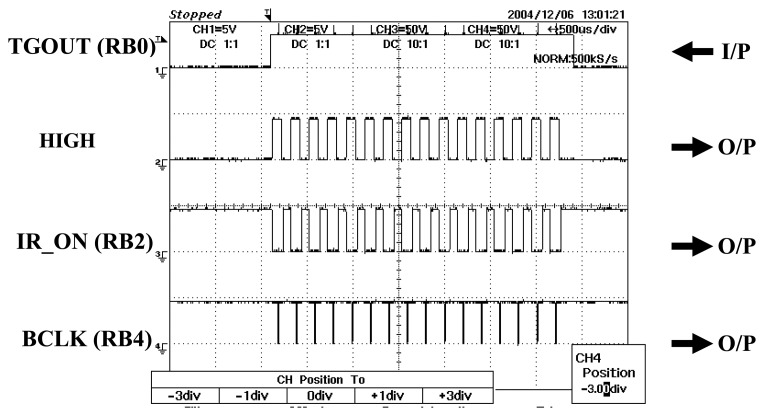
Microcontroller output signals in 2-projection mode (16 pulses).

**Figure 23.**
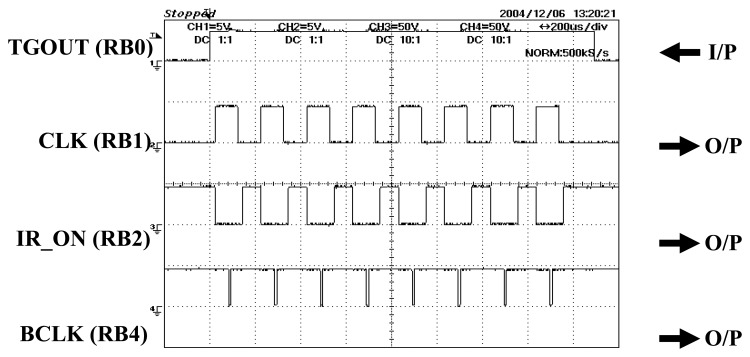
Microcontroller output signals in 4-projection mode (8 pulses).

Basically, the microcontroller remains in idle state (‘0’ state) until the PC sends a signal to request the DAS to start acquire data (‘1’ state). When the microcontroller is activated by the input signal, it will produce signals according to the programmed pulses. The PIC16F84A is programmed for two different modes for both the 2-projection mode and the 4-projection mode. In the 2-projection mode, the decoder requires 16 pulses to operate while the 4-projection mode needs only 8 pulses to function as shown in Figure 22 and Figure 23.

**Figure 24.**
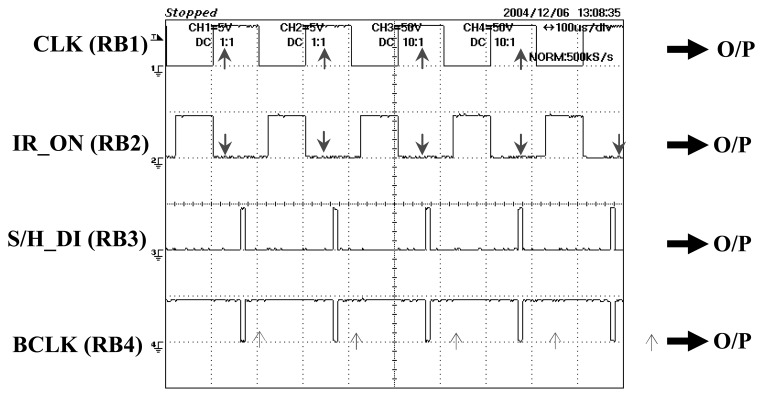
Timing and output control signals.

The CLK signal is the ‘heartbeat’ to the other control signals which is set at 5kHz in this research. The first rising edge of CLK will supply an ‘on’ pulse for emitter to start emitting light, as shown in Figure 24. As there are 32 transmitters used in two types of projection modes, the light sequence for one frame of light emission is tabulated in Table 1.

At the positive edge of the CLK signal too, the IR_ON will supply a negative edge trigger to the $\bar{G1}$ of the 74HC154 decoder (please refer to Figure 11) since $\bar{G1}$ is an active low pin. The decoder will stay activated every time the IR_ON signal is ‘0’ and after that deactivated when signal is ‘1.’

Meanwhile, the rising edge of S/H_DI will set S/H signal to ‘1’ to sample all output signals for a short period of 10μs and then continue to hold the sampled output signals until it receives the next rising edge. At the same time when the S/H signals are on hold, the BCLK signal will send a positive edge signal to the DAS to start data conversion. The total duration of the data conversion time depends on the burst mode clock frequency of 333 kHz in for this DAS. At the minimum sampling time of 3ms, the ideal conversion time would be 96μs; however due to the delays occurring while sending data to the DAS and the practical sampling time of 5μs, the conversion time is set at 390μs to ensure all data are converted properly.

#### 6.1.3 Output Voltages

**Figure 25.**
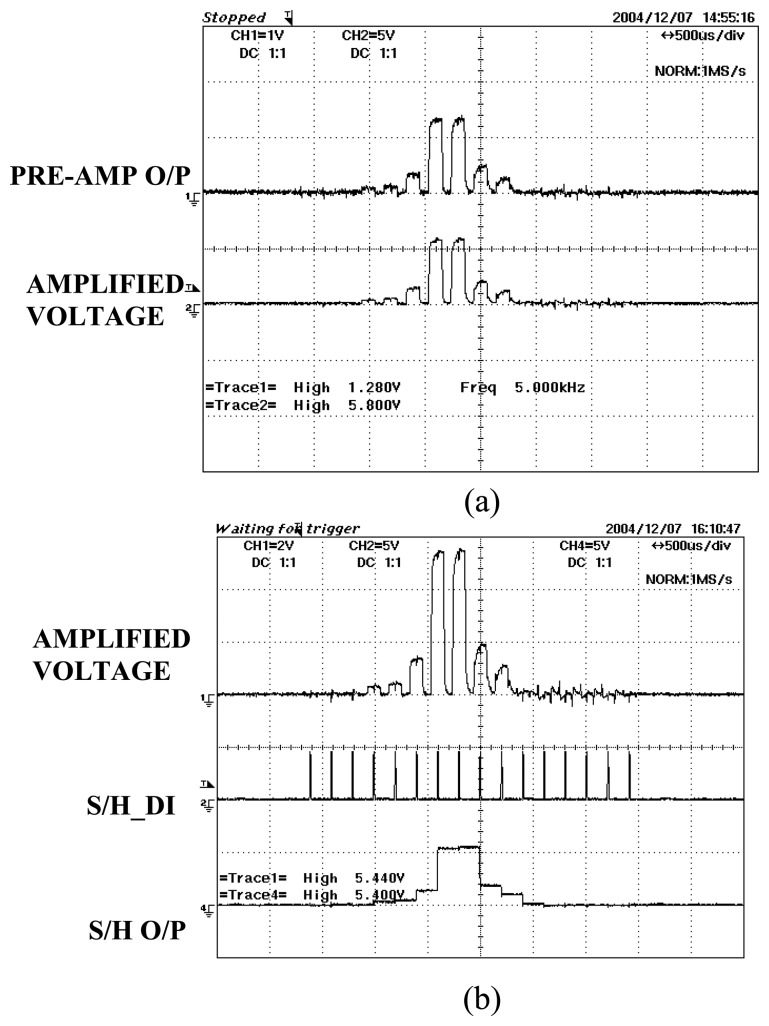
Output voltages for Rx23 in various stages **(a)** Pre-amp and amplification output signals **(b)** Amplified output signals, S/H_DI signal and output signals for S/H.

There are two levels of signal conditioning circuit, which are the pre-amp stage and the amplification stage. The output of the first stage usually consists of weak signals in the range of micro volts. These low level signals are then amplified with a certain gain until they are in the suitable range required for data conversion. The amplified output voltages will then be sent to the sample and hold. The digital control input from the microcontroller S/H_DI will drive the S/H to sample the waveform and then hold the sampled signal. These signals are then sent to the DAS for conversion. As an example, Figure 25 shows the pre-amp voltages, amplified voltages and sampled signals of Rx23 as an object passes through the sensing beam.

#### 6.1.4 Data Acquisition Rate (DAR)

The Data Acquisition Rate or DAR can be defined as the measurement of how fast the acquired signals are transferred from the hardware to the DAS in one frame. Basically, it can be explained in a simple manner according to Equation 5.

(5) $$\text{DAR}=\frac{1}{\text{Total Conversion Time}}$$

Whereby:

*DAR* = data acquisition rate in frames per second (unit fps).
*Total ConversionTime* = the total time needed to convert all the 32 receivers' signals in one frame (either in 2-projection or 4-projection mode).

**Figure 26.**
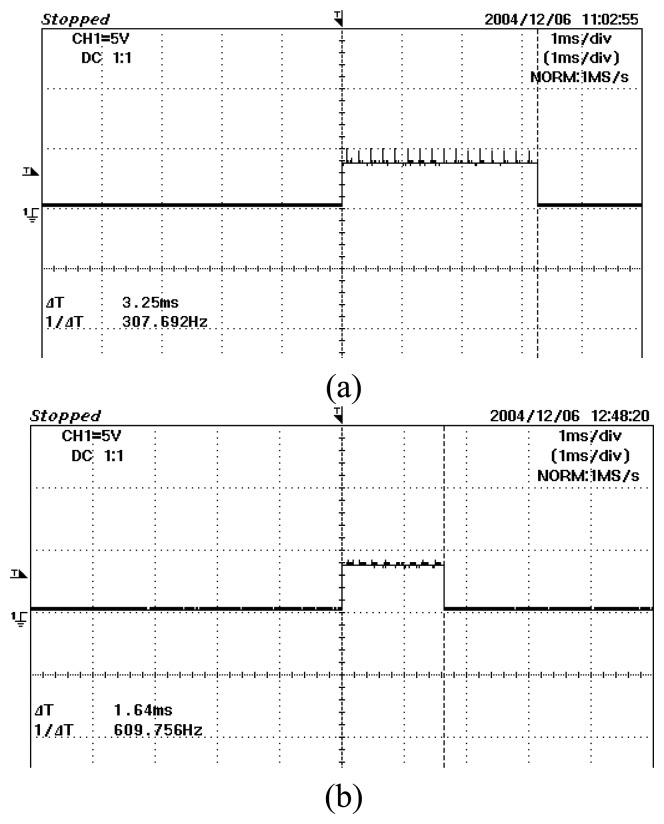
Total conversion time for one frame data **(a)** 2-projection **(b)** 4-projection.

The rising edge of TGOUT signal is generated from the DAS when user sends a signal to the DAS to start conversion. It remains at 5 volt until one frame of conversion process finishes. Thus, if we probe the TGOUT signal, we can measure the total conversion time for one frame of data. For a system which runs at 5 kHz in this research, the TGOUT signals probed for both the 2-projection and 4-projection modes are shown in Figure 26.

Based on Equation 6.1, the DAR obtained for both the projection modes are shown in Table 2.

It is proven here that the 4-projection mode has the ability to achieve higher DAR compared to the 2-projection mode. In the previous optical fan beam tomography research by Abdul Rahim [26], he used a total of 16 receivers with single projection each for 16 transmitters. He has managed to achieve a DAR of 300 fps. Theoretically, by using the conventional single projection technique with an increased number of sensors, the total time to convert one frame of data would be longer. It is known that a high DAR when acquiring data is essential in optical tomography system to prevent data loss.

**Figure 27.**
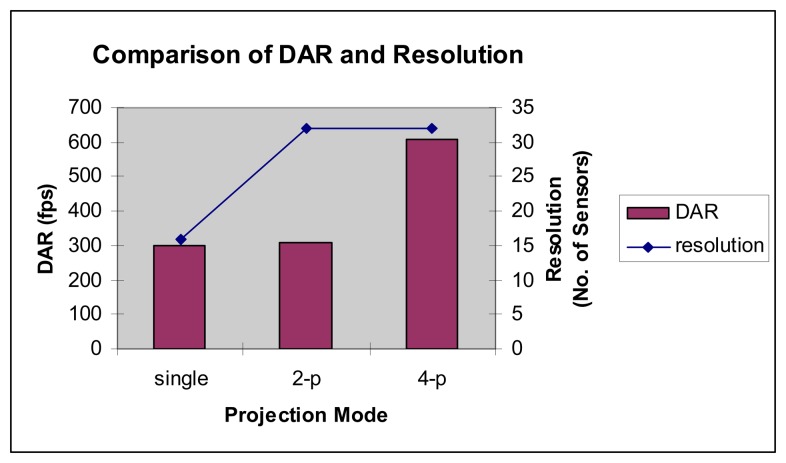
Comparison of DAR and resolution.

Thus, by comparing the number of sensors and DAR obtained by Chan [7] with the results achieved in this research, it has been verified that the multiple projection technique has a capability to increase the resolution of the hardware system (a higher number of sensors installed) and at the same time increasing the DAR (shorter time needed for data conversion in one frame). The graph shown in Figure 27 represents the improvement for the DAR achieved by multiple projection technique in this research when compared to the single projection result achieved by Chan [10].

In the graph, the resolution represents the number of sensors installed in the hardware system. The 2-projection technique spots an increase of 2.56% while the 4-projection technique shows an increase of about 103.25% in DAR compared to the previous research by Abdul Rahim [13].

## 7. Conclusions

This paper summarizes the hardware configuration and design for this project. To design the whole hardware, it is utmost important to take note on choosing the most suitable optical sensors, preparing the fibre optics and studying on the electronics and digital systems in order to design the associated circuits. PCB drawing skill must be acquired and the PIC instruction sets must be studied to enable source code writing and programming of PIC16F84A.

## Figures and Tables

**Table 1.** Light sequence for transmitters in one frame.

**CLK Pulse Number**	**Tx Group (2-projection mode)**	**Tx Group (4-projection mode)**
0	Tx0, Tx16	Tx0, Tx16, Tx8, Tx24
1	Tx1, Tx17	Tx1, Tx17, Tx9, Tx25
2	Tx2, Tx18	Tx2, Tx18, Tx10, Tx26
3	Tx3, Tx19	Tx3, Tx19, Tx11, Tx27
4	Tx4, Tx20	Tx4, Tx20, Tx12, Tx28
5	Tx5, Tx21	Tx5, Tx21, Tx13, Tx29
6	Tx6, Tx22	Tx6, Tx22, Tx14, Tx30
7	Tx7, Tx23	Tx7, Tx23, Tx15, Tx31
8	Tx8, Tx24	N/A
9	Tx9, Tx25	N/A
10	Tx10, Tx26	N/A
11	Tx11, Tx27	N/A
12	Tx12, Tx28	N/A
13	Tx13, Tx29	N/A
14	Tx14, Tx30	N/A
15	Tx15, Tx31	N/A

**Table 2.** DAR for different projection modes.

**Projection Mode**	**Total Conversion time**	**DAR**
2-projection	3.25 ms	307.69 fps
4-projection	1.64 ms	609.76 fps

## References

[1] Williams R.A., Beck M.S. (1995). Process Tomography: Principles, techniques and applications.

[2] West R.M., Jia X., Williams R.A. (1999). Parametric Modelling in Industrial Process Tomography. 1^st^World Congress on Industrial Process Tomography.

[3] Ruzairi A.R. (1996). A Tomography Imaging System for Pneumatic Conveyors Using Optical Fibres. Ph.D. Thesis.

[4] Sallehuddin I. (2000). Measurement of Gas Bubbles in a Vertical Water Column Using Optical Tomography. Ph.D. Thesis.

[5] Khoo B.F. (2002). Optical Fibre Sensors for Process Tomography. B.Sc. Thesis.

[6] Hisyamuddin S. (2001). Sistem Tomografi Optik Berkejituan Tinggi. B.Sc. Thesis.

[7] Chan K.S. (2002). Time Image Reconstruction for Fan Beam Optical Tomography System. M.Sc. Thesis.

[8] Pang J.F. (2004). Real Time Image Reconstruction for Fan Beam Optical Tomography System. M.Sc. Thesis.

[9] Welch H.L. (2000). Phototransistor. EE210-S00-Lecture.

[10] Chan K.S., Ruzairi A.R. (2002). Tomographic Imaging of Pneumatic Conveyor Using Optical Sensor. Proceeding 2^nd^World Engineering Congress in Malaysia.

[11] Van Eijkelenborg M.A., Large M.C.J., Argyros A., Zagari J., Manos S., Issa N.A., Bassett I., Fleming S., McPhedran R.C., de Sterke C.M., Nicorovici N.A.P. (2001). Microstructured Polymer Optical Fibre. Optics Express.

[12] Syms R., Cozens J. (1992). Optical Guided Waves and Devices.

[13] Abdul Rahim R., Chan K.S., Pang J.F., Leong L.C. (2005). Optical Tomography System Using Switch-Mode Fan Beam Projection: Modelling Techniques. Journal Optical Engineering.

[14] (1995). Burr-Brown. Photodiode Monitoring with Op-Amps. Application Bulletin.

[15] Wong N. (2001). CMOS Integrated Photodiode and Low Voltage Transimpedance.

[16] Philips Semiconductors (2004). 74HC154 4-16 Line Decoder/Demultiplexer.

[17] Pang J.F., Abdul Rahim R., Chan K.S. (2004). Real Time Image reconstruction System Using Two Data Processing Unit in Optical Tomography.

[18] Texas Instruments (2003). SN54HC04 HEX Inverters.

[19] Tocci R.J., Widmer N.S. (1998). Digital Systems: Principles and Applications.

[20] Hallmark C.L., Horn D.T. (1991). The Master IC Cookbook.

[21] Philips Semiconductors (1990). 74HC4016 Quad Bilateral Switches.

[22] Jung W. (1995). Applying IC Sample-Hold Amplifiers.

[23] National Semiconductor Corporation (1995). Monolithic Sample-and-Hold Circuits.

[24] National Semiconductor Corporation Circuit Applications of Sample-Hold.

[25] Leonard B. (1993). Picking the Right Sample-And-Hold Amp for Various Data-Acquisition Needs.

[26] Abdul Rahim R., Chan K.S. (2004). Optical Tomography System for Process Measurement Using LED as a Light Source. Journal Optical Engineering.

